# Household Transmission of Pandemic (H1N1) 2009 Virus, Taiwan

**DOI:** 10.3201/eid1710.101662

**Published:** 2011-10

**Authors:** Luan-Yin Chang, Wei-Hua Chen, Chun-Yi Lu, Pei-Lan Shao, Tsui-Yien Fan, Ai-Ling Cheng, Li-Min Huang

**Affiliations:** Author affiliation: National Taiwan University, Taipei, Taiwan

**Keywords:** influenza virus subtype H1N1, pandemic (H1N1) 2009, household transmission, outcome, virus, viruses, influenza, Taiwan, dispatch

## Abstract

During August–November 2009, to investigate disease transmission within households in Taiwan, we recruited 87 pandemic (H1N1) 2009 patients and their household members. Overall, pandemic (H1N1) 2009 virus was transmitted to 60 (27%) of 223 household contacts. Transmission was 4× higher to children than to adults (61% vs. 15%; p<0.001).

Pandemic (H1N1) 2009 was first identified in 2 southern California counties in April 2009 (*1*), and the World Health Organization declared a global pandemic on June 11, 2009 (*2*). In Taiwan, the government suggested that persons with pandemic (H1N1) 2009 remain home until 24 hours after they were symptom free (*3*). In some influenza epidemics, ≈50% of households have >1 members who become infected (*4*). Further investigation into the transmission of pandemic (H1N1) 2009 virus among household members is needed to help control and prevent additional infections. We investigated the transmission of pandemic (H1N1) 2009 virus and clinical outcomes of infection within households of persons with laboratory-confirmed infection.

## The Study

During August–November 2009, we enrolled patients at the National Taiwan University Hospital who were infected with pandemic (H1N1) 2009 virus and their household members. The following samples were obtained from patients with clinical signs and symptoms suggestive of pandemic (H1N1) 2009 infection who visited the emergency department, outpatient clinics, or inpatient wards: nasopharyngeal swab specimen for rapid influenza antigen testing (QuickVue A+B test; Quidel, San Diego, CA, USA), throat swab specimen for virus isolation and novel subtype H1N1 reverse transcription PCR (RT-PCR), and blood specimen for serum hemagglutination inhibition (HI) assays. Laboratory-confirmed pandemic (H1N1) 2009 infection was defined in 3 ways: 1) isolation of influenza A virus, followed by positive RT-PCR result for pandemic (H1N1) 2009 virus; 2) positive rapid influenza A test result, followed by positive RT-PCR result for pandemic (H1N1) 2009 virus; or 3) pandemic (H1N1) 2009 virus HI titer >40. None of the participants had received an influenza subtype H1N1 vaccine before this study.

Persons with laboratory-confirmed pandemic (H1N1) 2009 and their household members were sent a letter and/or received a telephone call inviting them to participate (Figure). After they accepted the invitation, we collected their case report forms, which contained data regarding the source of infection, final diagnosis, clinical manifestations, and course of the disease. The index patient was defined as the first person in a household to have laboratory-confirmed pandemic (H1N1) 2009 (body temperature >38.0°C and/or cough and/or sore throat). All enrolled index patients and their household members provided blood samples for further HI assays. A mean of 45 days (SD 26, median 36, range 12–107 days) elapsed between the first day of illness in the index patient and household investigations, including blood sampling. The household transmission rate (secondary attack rate) was defined as the percentage of household members who had laboratory-confirmed pandemic (H1N1) 2009 infection 1–7 days after the onset of symptoms in the index patient.

**Figure:**
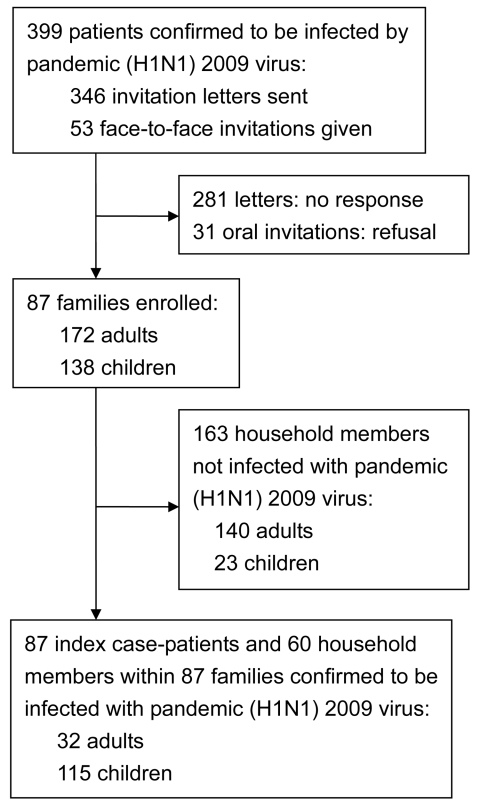
Flowchart showing household transmission of pandemic (H1N1) 2009 virus infection, Taiwan, August–November 2009.

During August–November 2009, pandemic (H1N1) 2009 was confirmed for 399 patients at National Taiwan University Hospital. Of those 399 patients, 87 patients and their households were enrolled in the study; households included the 87 index patients and their 223 household contacts (172 adults and 138 children) (Figure). Of the 87 index patients, 72 (83%) had visited the hospital for illness and had laboratory-confirmed pandemic (H1N1) 2009 infection (PCR-positive test results, HI titer >40, or both); the remaining 15 (17%) index patients attended community clinics and were identified by having titers >40 for pandemic (H1N1) 2009 virus on HI test when household investigations were done. The possible source of infection was traced for 52 (60%) of the 87 households: 46 (53%) infections were traced to schools, 5 (6%) infections were traced to daycare centers or other child care situation (when 1 babysitter cared for a few children), and 1 (1%) infection was traced to a summer camp.

As shown in Table 1, the mean ages of the index patients and their household contacts were 10.6 and 33.8 years, respectively; only 6 (7%) of the 87 index patients were adults. Households contained a mean of 1.9 children (SD 0.8, median 2, range 0–4 children).

**Table 1:** Demographic characteristics and pandemic (H1N1) 2009 infection rates among 223 household contacts of 87 index case-patients, by contact type, Taiwan, August–November 2009*

Study participants	No. participants	Mean age, y (SD)	Sex, F/M	Positive for pandemic (H1N1) 2009		No. positive/no. tested (%)
				By PCR, no.	By HI, no./no. tested (%)	
Index case-patients	87	10.6 (7.2)	42/45	71	81/84 (96)	87/87 (100)
Household members	223	33.8 (17.9)	122/101	21	59/222 (27)†	60/223 (27)
Children	57	8.0 (3.6)	28/29	21	34/56 (61)†	35/57 (61)
Siblings	56	7.9 (3.6)	28/28	21	34/55 (62)†	35/56 (63)
Cousin	1	11.5	0/1	0	0/1 (0)	0/1 (0)
Adults	166	43.2 (10.5)	94/72	0	25/166 (15)	25/166 (15)
Siblings	5	20.3 (1.7)	3/2	0	0/5 (0)	0/5 (0)
Parents	138	41.0 (5.6)	76/62	0	20/138 (14)	20/138 (14)
Grandparents	18	66.6 (5.9)	11/7	0	4/18 (22)	4/18 (22)
Uncles/aunts	5	41.5 (1.7)	4/1	0	1/5 (20)	1/5 (20)
Total	310	27.5 (18.9)	169/141	92	140/310 (45)	147/310 (47)

*HI, hemagglutination inhibition. †One person was confirmed to be infected with pandemic (H1N1) 2009 virus by PCR of a throat swab specimen without testing of a blood sample.

Pandemic (H1N1) 2009 virus was transmitted to 60 (27%) of the 223 household contacts. The virus was transmitted to 35 (63%) of 56 child-aged siblings (but not to 1 cousin), to none of 5 adult-aged siblings, to 20 (14%) of 138 parents, to 4 (22%) of 18 grandparents, and to 1 (20%) of 5 aunts and uncles. Percentage of transmission among the different groups of household contacts differed significantly: the virus was transmitted to 35 (61%) of the 57 children and to 25 (15%) of the 166 adults (p<0.01 by χ^2^ test). However, percentage of transmission among different adult groups did not differ significantly (p = 0.86 by χ^2^ test). Mean interval between the onset of illness in the index patient and household members was 3.3 days (SD 2.6, median 3, range 1–6 days).

Of the 147 patients with pandemic (H1N1) 2009, 119 (81%) received a diagnosis of influenza-like illness; 10% received a diagnosis of upper respiratory tract infection; 3% each received a diagnosis of bronchitis, bronchopneumonia, asthma, or acute gastroenteritis; and 2% received a diagnosis of pneumonia. Of the 147 patients (all children), 10 (7%) were hospitalized and discharged without sequelae. Seventy-seven (89%) of the 87 index patients and 29 (48%) of the 60 household members received oseltamivir.

Table 2 shows attack rates and odds ratios for pandemic (H1N1) 2009 virus infection among the 223 household contacts by patient characteristics (sex and age) and signs and symptoms. Age <18 years, fever, cough, sore throat, rhinorrhea, myalgia, and malaise were significantly associated with pandemic (H1N1) 2009 infection, but age <18 years, fever, and cough most significantly predicted the transmission of pandemic (H1N1) 2009 virus in multivariate analysis with a multiple logistic regression model. However, we did not find a significant relationship between index patient characteristics, specific symptoms, lower respiratory tract infection, or the need for hospitalization and the rate of household transmission of pandemic (H1N1) 2009 virus.

**Table 2:** Pandemic (H1N1) 2009 attack rates among 223 household contacts of 87 index patients, by patient characteristics and symptoms, Taiwan, August–November 2009*

Characteristic	Attack rate, %	OR (95% CI)†	p value†
Sex			0.21
M, n = 101	23	Reference	
F, n = 122	30	1.48 (0.81–2.70)	
Age, y			<0.0001
>18, n = 166	15	Reference	
<18, n = 57	61	9.09 (4.55–17.86)	
Signs and symptoms			
Fever			<0.0001
No, n = 163	12	Reference	
Yes, n = 60	68	16.13 (7.87–33.33)	
Cough			<0.0001
No, n = 158	13	Reference	
Yes, n = 65	60	10.42 (5.29–20.83)	
Rhinorrhea			<0.0001
No, n = 176	19	Reference	
Yes, n = 47	55	5.18 (2.60–10.31)	
Sore throat			0.0002
No, n = 176	21	Reference	
Yes, n = 47	49	3.60 (1.83–7.09)	
Vomiting			0.06
No, n = 214	26	Reference	
Yes, n = 9	56	3.60 (0.93–13.90)	
Diarrhea			0.23
No, n = 214	26	Reference	
Yes, n = 9	44	2.30 (0.60–8.85)	
Malaise			0.002
No, n = 200	24	Reference	
Yes, n = 23	57	4.20 (1.73–10.20)	
Myalgia			0.02
No, n = 192	24	Reference	
Yes, n = 31	45	2.61 (1.20–5.71)	

*OR, odds ratio; CI, confidence interval. †Univariate logistic regression model was used for unadjusted OR (95% CI) and unadjusted p values. If features were significantly different with p<0.05 in univariate logistic regression model, they were further analyzed with multiple logistic regression model for adjusted OR (95% CI) and adjusted p values.

## Conclusions

We found children to be >4× more susceptible than adults to the secondary transmission of pandemic (H1N1) 2009 virus within households (61% vs. 15%). Furthermore, 93% of our index patients were children, and for ≈60% of them, the source of exposure to the virus was a school or daycare center. Thus, children play major roles in the introduction and spread of influenza within families. Vaccination and other measures will prevent susceptible children from becoming infected and reduce influenza virus transmission among families and communities.

This study has limitations, however, for example, the potential for nonresponse bias and possible preferential recruitment of families with sick children as index patients. Thus, adults may be relatively underrepresented as index patients in this study. Also, some adults may be less likely to go to the hospital with influenza-like symptoms.

In our study, the secondary attack rate in households was 27%, which is similar to rates in studies by Komiya et al. (26%), Sikora et al. (30.2%), and Looker et al. (33%) but higher than rates in studies by Cauchemez et al. (13%) and Carcione et al. (14.5%) (*5**–**9*). The secondary attack rate found in this study may have been relatively high because, without a vaccine against pandemic (H1N1) 2009, there were more susceptible children in the households and because most index patients were children who may shed virus for a longer period (*10*). Our findings show the key role that children play in introducing and spreading pandemic (H1N1) 2009 virus within households. Public health measures, such as vaccination and community health education, can prevent infections among children and help reduce virus transmission among families and the larger community.
